# The Neurotropic Parasite Toxoplasma Gondii Increases Dopamine Metabolism

**DOI:** 10.1371/journal.pone.0023866

**Published:** 2011-09-21

**Authors:** Emese Prandovszky, Elizabeth Gaskell, Heather Martin, J. P. Dubey, Joanne P. Webster, Glenn A. McConkey

**Affiliations:** 1 Institute of Integrative and Comparative Biology, Faculty of Biological Sciences, University of Leeds, Leeds, United Kingdom; 2 Animal Parasitic Diseases Laboratory, USDA, ARS, ANRI, BARC-East, Beltsville, Maryland, United States of America; 3 Department of Infectious Disease Epidemiology, Faculty of Medicine, Imperial College, London, United Kingdom; Instituto Butantan, Brazil

## Abstract

The highly prevalent parasite *Toxoplasma gondii* manipulates its host's behavior. In infected rodents, the behavioral changes increase the likelihood that the parasite will be transmitted back to its definitive cat host, an essential step in completion of the parasite's life cycle. The mechanism(s) responsible for behavioral changes in the host is unknown but two lines of published evidence suggest that the parasite alters neurotransmitter signal transduction: the disruption of the parasite-induced behavioral changes with medications used to treat psychiatric disease (specifically dopamine antagonists) and identification of a tyrosine hydroxylase encoded in the parasite genome. In this study, infection of mammalian dopaminergic cells with *T. gondii* enhanced the levels of K+-induced release of dopamine several-fold, with a direct correlation between the number of infected cells and the quantity of dopamine released. Immunostaining brain sections of infected mice with dopamine antibody showed intense staining of encysted parasites. Based on these analyses, *T. gondii* orchestrates a significant increase in dopamine metabolism in neural cells. Tyrosine hydroxylase, the rate-limiting enzyme for dopamine synthesis, was also found in intracellular tissue cysts in brain tissue with antibodies specific for the parasite-encoded tyrosine hydroxylase. These observations provide a mechanism for parasite-induced behavioral changes. The observed effects on dopamine metabolism could also be relevant in interpreting reports of psychobehavioral changes in toxoplasmosis-infected humans.

## Introduction

A complex range of interactions exist between a pathogen with its host, which may include manipulation of the host for the pathogen's own advantage. There are several examples of viruses, such as rabies virus [1], and parasites, including *Acanthocephala* spp. [2] and *Toxoplasma gondii* [3], that influence host behavior to increase their transmission efficiency. For years, scientists have been intrigued by the association between *T. gondii* infection and altered aversive behavior. The underlying mechanism(s) responsible for this behavior change are presently unknown. The aim of our study was to identify a possible explanation for this phenomenon.

*T. gondii* is a common, global protozoan parasite, which requires both a definitive host and an intermediate host to complete its life cycle. Although felines are the only definitive host of *T. gondii*, any warm-blooded animal, including humans, can be infected [4]. It is estimated that one quarter of the population (over 12 years of age) in the United States is positive for *T. gondii* infection (Center for Disease Control, USA, 2008). Prevalence in some areas can be as high as 95% in older populations. Latent, chronic infection, which is characterized by parasite encystment in the host muscle and brain cells (particularly neurons and glial cells), persists following the resolution of acute infection and continues with seropositivity throughout the host's lifetime [4]. Due to its high prevalence in the human population, it is critical to better understand the effects of *T. gondii* infection in the brain.

During the chronic stage of infection, infected rodents, which are a key intermediate host for *T. gondii*, exhibit a distinct repertoire of specific behavioral changes, including a loss of aversion to cat odors [3], [5]. Infected rodents are, conversely, attracted to these odors, and this may be responsible for increased predation and for an increase in successful transmission of the parasite to the feline host; as cats are the only animal that can shed the environmentally-resistant stage of the parasite known as oocysts. This behavior change in infected rodents during the chronic stage of infection appears highly specific to feline odor, as a similar change is not evoked by other predators and has no effect on conditioned fear and anxiety [6], [7]. The underlying mechanism(s) responsible for this behavior change still remain unclear, however, it has been revealed that anti-psychotic (haloperidol) and mood-stabilizing medication (valproic acid) can prevent the development of these behavior changes [7] in addition the dopamine uptake inhibitor GBR12909 modifies behavioral responses associated with latent toxoplasmosis in infected rodents [8]. Furthermore, we have recently identified an enzyme with tyrosine hydroxylase activity encoded in the *Toxoplasma* genome whose expression is induced during differentiation to tissue cyst stages [9]. Several studies have suggested that *T. gondii* infection in humans can have serious neurological effects [10]. Associations have been identified between *T. gondii* seroprevalence and schizophrenia [11]–[13]. The schizophrenia-associated risk factors of *T. gondii* infection have been found to be greater than the risk factors associated with an individual's genes and with other environmental factors [13], [14]. Schizophrenia affects approximately 1% of the adult population and in most cases is a lifelong disease with exacerbations. Although schizophrenia is a multifactorial disease, pharmacological and genetic evidence suggest that dysregulation of dopamine metabolism is involved in schizophrenia [15], [16].

Thus, it is crucial to examine whether dopamine metabolism is affected by *T. gondii* infection, particularly based on evidence of a tyrosine hydroxylase encoded by *T. gondii*. To address these questions, dopamine metabolism was monitored *in vivo* in the brains of chronically infected mammals and monitored *in vitro* during infection of neural cells.

## Methods

### Ethics statement

All animal work was performed according to national and international guidelines following approved animal procedures by the Beltsville Area Animal Care Committee, United States Department of Agriculture (Protocol no. 09-010–Toxoplasmosis in mice; approved June 4, 2009). This protocol is reviewed annually, and any amendments are approved separately.

### Growth of parasites and host cells

*T. gondii* strains were maintained in human foreskin fibroblasts (HFFs) as previously described [9]. PC-12 cells obtained from ECACC (Salisbury) were maintained as described by the supplier.

### Mouse strains

Female Swiss Webster mice infected with *T. gondii* VEG strain were used for histology.

### Immunofluorescence assay of brain sections

Immunofluorescence against multiple targets was performed on paraformaldehyde-fixed, paraffin-embedded mouse brain sections. Female Swiss Webster mice were infected with *T. gondii* VEG strain oocysts 6–8 weeks prior to processing. Tissues were collected, formalin-fixed and paraffin-embedded using standard protocols and following approved guidelines. Slides were deparaffinized and rehydrated with an alcohol descending row, which was then followed by epitope retrieval in 10 mM sodium citrate buffer (pH 6.0) overnight at 60°C following sectioning. Slides were blocked with 2% normal goat sera for 1 h at room temperature. TRITC-conjugated lectin from *Dolichos biflorus* (Cat # L9658, Sigma, St. Louis) was introduced to the slides for 4 h at room temperature, diluted 1∶200 in primary staining solution (1% BSA, 0.1% cold fish skin gelatine, 0.5% Triton X-100 in 0.1 M PBS pH 7.2). Next, samples were washed (3×10 min) in wash buffer (TBS pH 8.4 with 0.1% Triton X-100 and 1% fish skin gelatin) and blocked using a biotin-streptavidin blocking kit (Cat # SP-2002, Vector Labs, Peterborough) according to the manufacturer's protocol. Samples were incubated with primary antibody (raised in rabbit) against dopamine (Cat # ab8888, Abcam, Cambridge, MA) (diluted 1∶200) or tyrosine hydroxylase (Cat # ab112, Abcam) (diluted 1∶500) overnight at 4°C. Samples were rinsed with wash buffer and incubated for 1 h with biotinylated anti-rabbit IgG secondary antibody (Cat # B-1000, Vector Labs) diluted 1∶500 in secondary antibody solution (0.05% Tween in 0.1 M PBS pH 7.2). Sections were rinsed and incubated with FITC-conjugated streptavidin (Cat # SA-5001, Vector Labs) diluted 1∶100 according to the manufacturer's guidelines in secondary antibody buffer at room temperature. Finally, slides were rinsed in wash buffer containing DAPI and double-distilled water prior to mounting in Fluoromount G (Southern Biotech, Birmingham). All incubation and blocking steps were carried out in a wet chamber. All slides were kept at 4°C in the dark before imaging using a Zeiss LSM510 META laser scanning inverted AxioVert 200M confocal microscope with DIC optics. 3D reconstructions of serial sections were generated with the same equipment using the LSM imaging software for the 3D deconvolution. To assess the specificity of dopamine staining, sections were incubated either without primary antibody or with primary anti-dopamine antibody in the presence of freshly prepared dopamine or serotonin for 30 min prior to and for overnight following addition to the sections.

A *T. gondii* tyrosine hydroxylase antibody custom antibody (Genscript, Piscataway) was developed to assess the parasite enzyme in animals. The affinity purified antibody is directed against a unique sequence (CIRSSPDPLDLRKMT) in the amino terminal domain that is not found in mammalian tyrosine hydroxylase and has no significant similarity to any protein in the predicted mammalian proteome or other proteins of the *T. gondii* proteome. The specificity of the antibody for *T. gondii* tyrosine hydroxylase was confirmed by Western analysis. Total protein from half mouse brains was isolated in 20 volumes (wt/vol)lysis buffer (20 mM Tris-HCl pH 8; 137 mM NaCl; 10% glycerol; 1% Triton X; 2 mM EDTA and protease inhibitors (cOmplete Mini EDTA-free cocktail, Roche)) and quantified using Bradford reagent (Sigma) as per manufacturer's instructions. Expression and purification of *T. gondii* tyrosine hydroxylase was as previously described [9]. SDS-PAGE was following standard protocols with 2–20 µg protein separated on a 12% sodium dodecylsulphate- polyacrylamide gel. The proteins were transferred to nitrocellulose membrane, blocked with 5% non-fat dried milk in PBS containing 0.05% Tween-20 (vol/vol) for 1 hour. Incubation with the custom antibody (1∶2500) 4°C overnight was followed by washing in PBS-Tween (0.05%) and incubation with an anti-rabbit (1∶5000) conjugated horseradish peroxidase antibody (Sigma) at room temperature for 1 hour. Blots were then washed as above and developed using Supersignal West Pico Chemiluminescent kit (Pierce, Rockford, IL). Bands were visualised with an X-Omat film system. The membrane was stripped and re-probed with mouse anti-β-actin (1∶25,000; Sigma) overnight at 4°C followed by anti-mouse (1∶10,000) conjugated horseradish peroxidase antibody (AutogenBioclear, Wiltshire, UK) at room temperature for 1 hour and subsequent visualisation. The anti-*T. gondii* tyrosine hydroxylase antibody was used for immunofluorescence (diluted 1∶500) following a similar protocol as described above.

Immunofluorescence of tyrosine hydroxylase in cultured parasites was performed with paraformaldehyde-fixed cell cultures. Cultures of *T. gondii* stably expressing RFP-conjugated GRASP protein (kindly donated by Manami Nishi from David Roos laboratory, University of Philadelphia, USA) in human foreskin fibroblasts grown on polylysine-coated coverslips were alkaline induced for differentiation as published and differentiation monitored by counting the number of parasites in the vacuoles in the normal and pH shifted cultures. These conditions yielded bradyzoite forms as shown by RT-qPCR with the bradyzoite markers BAG1 and SAG4 [9]. After five days, coverslips were paraformaldehyde-fixed and probed with tyrosine hydroxylase antibody (Cat # ab112, Abcam) (diluted 1∶500) with visualisation as described above.

### Immunohistochemical assay of brain sections

For immunohistochemical assays, sections were treated as described above, except for the following steps: for washing and dilution buffers, 0.1 M PBS supplemented with 0.1% Tween was used. After antigen retrieval, slides were incubated in 0.3% H_2_O_2_ (in 0.1 M PBS) to quench endogen peroxidases. Following secondary antibody treatment, 5 µg/ml HRP-conjugated streptavidin (Cat # SA-5004, Vector Labs) was applied, and next, the peroxidase substrate kit (Vector Labs, ImmPACT™DAB, Cat # SK-4105) was used according to the manufacture's protocol. Prior to mounting, sections were stained with haematoxylin to visualize cell nuclei. Imaging of slides was performed using a Zeiss Axioplan microscope equipped with DIC optics. Photomicrographs were collected with a Photometrics CoolSNAP camera and Improvision Openlab software.

### Glyoxylic acid staining

A cytochemical method was used to assess the dopamine staining of tissue cyst-containing neural cells in infected mice brain. Glyoxylic acid reacts with catecholamines in a gaseous reaction to form fluorescent products. Dopamine reacts with glyoxylic acid to form a product that specifically emits at 478–480 nm [17].

### Dopamine accumulation and release from dopaminergic cells

The dopaminergic cell line PC12 (ECACC) was infected with Prugniard strain of *T. gondii* tachyzoites that had been alkaline shocked to induce bradyzoite differentiation. Liberated tachyzoites were incubated in RPMI media at pH 8 with 1% FCS at 37°C and ambient CO_2_ for 16–18 h, then rinsed with DMEM and returned to standard PC12 cell culturing conditions. PC12 cultures were infected 2.5×10^5^–7.5×10^5^ parasites and cultured for five days prior to assay. The cultures infected with higher numbers of parasites had parasitemia of 40–50%. Prior to assay, samples were normalized to equivalent numbers of cells (2.5×10^6^) per assay. One set of cultures was harvested by centrifugation and lysed by sonication in 0.1 M perchlorate for total dopamine measurement by HPLC with electrochemical detection. A parallel set of cultures were equilibrated with wash buffer with low KCl containing buffer (140 mM NaCl, 4.7 mM KCl, 1.2 mM MgCl_2_, 2.5 mM CaCl_2_, 11 mM dextrose, 10 mM HEPES, pH 7.4) for 30 min followed by incubation with two volumes high KCl containing buffer (40 mM NaCl, 100 mM KCl, 1.2 mM MgCl_2_, 2.5 mM CaCl_2_, 11 mM dextrose, 10 mM HEPES, pH 7.4) for 2 min to induce dopamine release as previously described [18]. During the assay, samples were taken from the media, washing buffer, and high KCl containing buffer and immediately supplemented with 0.3 volumes 0.1 M perchlorate. Three independent experiments were performed with a representative experiment shown.

Following centrifugation, cell homogenates and media were assayed by HPLC-ED. Reverse phase chromatography, combined with electrochemical detection, was performed with a Dionex HPLC system consisting of a P580 Pump (Dionex) and Ultimate 3000 Autosampler Column Compartment with a C18 Acclaim 120 column (5 µm, 4.6×150 mm) and an ESA Coulochem III cell, equipped with a glassy carbon electrode used at 700 mV versus Ag/AgCl reference electrode for detection of monoamines. The mobile phase consisted of degassed 57 mM anhydrous citric acid (Fisher Scientific, Loughborough), 43 mM sodium acetate (Dionex, Sunnyvale) buffer containing 0.1 mM EDTA (Sigma Aldrich), 1 mM sodium octanesulphonate monohydrate, and 10% methanol. The pH was adjusted to 4. The mobile phase was delivered at a flow rate of 1.5 ml/min, and the column temperature was set at 40°C. Applied standards (dopamine, L-DOPA) were dissolved in 0.1 M perchlorate for chromatography. The concentration of compounds was determined using Chromeleon software.

## Results

### Dopamine metabolism in infected neural cells in brain tissue

**Figure 1:**
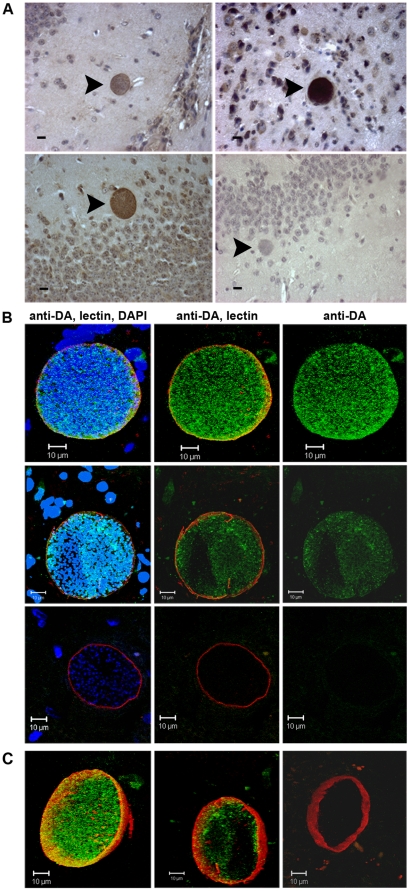
Dopamine in tissue cysts of *T. gondii* in brain tissue sections. (A) Dopamine was detected in brain tissue sections of chronically infected Swiss Webster mice by immunohistochemical staining with anti-dopamine antibody and horseradish peroxidase. Tissue cysts containing hundreds of bradyzoites are visible as brown circular structures (arrowheads) in infected brains. The bottom right panel is a control lacking anti-dopamine antibody. All black bars are 10 µm long. (B) Localization by indirect immunofluorescence of brain sections stained with anti-dopamine antibody (green), DAPI (blue), and TRITC-lectin (red). Three sections are shown from different regions of the brain in the top, middle and bottom rows of panels with the negative control (no primary antibody) in the bottom row. In each series all three channels are illuminated (left), the anti-dopamine and lectin channels are illuminated (center), and only the anti-dopamine channel is illuminated (right). The DAPI identifies neural cells and the individual bradyzoites within the tissue cyst and the lectin stains the surface of the cyst. The dopamine staining appeared specific (also see Fig. 2) as the antibody stained neurons in the striatum, amygdala and hippocampus. (C) A 3D projection of a Z-stack reconstruction of serial images of a tissue cyst within a brain section stained with anti-dopamine antibody and lectin as described in B. Control without the primary anti-dopamine antibody is shown in the right panel.

**Figure 2:**
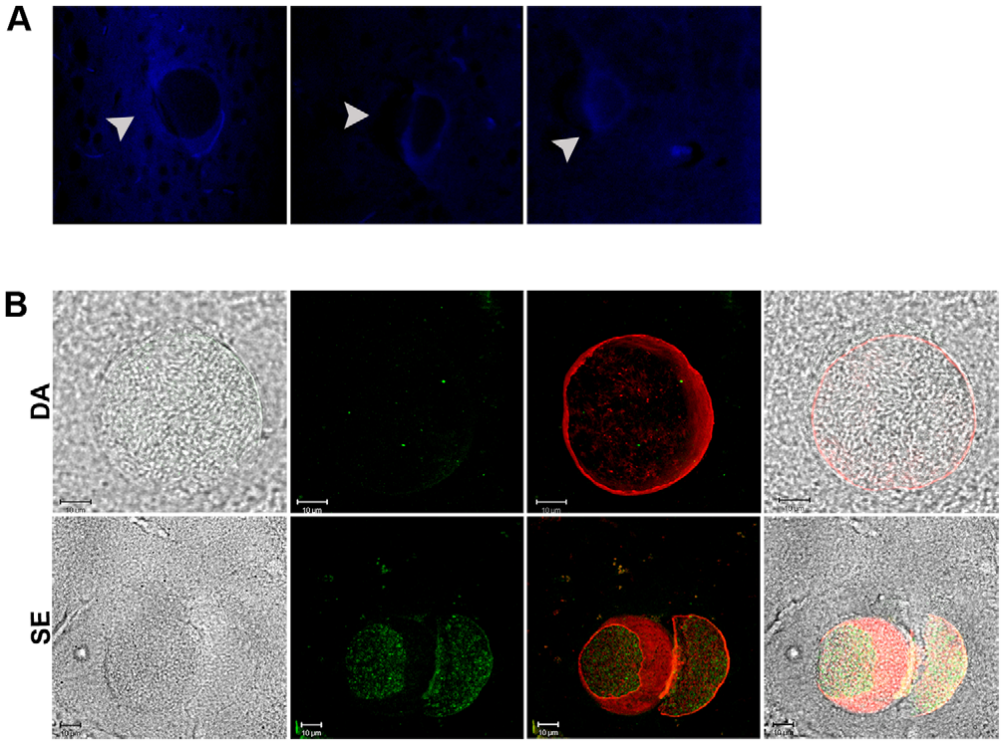
Specificity of dopamine *staining T. gondii* tissue cysts. (A) Histochemical (glyoxylic acid) staining of dopamine in brain sections from chronically-infected mice detected by fluorescence. Glyoxylate reacts with dopamine to fluoresce blue-white [17]. Cells containing *T. gondii* cysts in brain tissue exhibited blue-white fluorescence. The tissue cysts stained darkly, similar to mouse cell nuclei, presumably due the high density of bradyzoites. (B) Brain tissue sections from chronically-infected mice were stained with indirect fluorescein staining as in Fig. 1 except the anti-dopamine primary antibody was incubated in the presence of 50 µg/ml dopamine (top) and 50 µg/ml serotonin (botto). From left to right: bright field, fluorescein only channel (green), fluorescein and lectin-TRITC (green and red channels, respectively), and both channels plus bright field. Serotonin did not compete for dopamine staining.

A previous study found that the global content of dopamine in the brains of mice chronically infected with *T. gondii* was increased by 14% (114% of uninfected (P<0.01)), whereas other neurotransmitters were unchanged [19]. The localized effects of *T. gondii* infection on dopamine metabolism in tissue cysts have not been examined. *T. gondii* forms intracellular tissue cysts in neurons with each tissue cyst containing hundreds of bradyzoites (slowly dividing stage) that may remain *in situ* through the host's lifetime [4]. Formaldehyde-fixed brain sections from mice chronically infected with *T. gondii* were probed with dopamine antibody (Abcam). Dopamine antibody staining was readily apparent in infected cells (Fig. 1). Surprisingly, the localization was primarily within the *T. gondii* tissue cysts containing the parasites visualized as intensely stained cysts (Figs. 1, 2), rather than the host neural cell. The dopamine antibody staining in tissue cysts was punctate. Image rotation illuminated staining throughout the tissue cyst with most concentrated staining near the periphery. The antibody was raised against dopamine glutaraldehyde conjugated to bovine serum albumin (BSA). The antibody also labelled neurons in the amygdala and hippocampus in uninfected and infected mice (data not shown); areas with a high concentration of dopaminergic neurons. Intracellular tissue cysts were identified based on morphology (for immunohistochemistry) and by labeling the periphery of the tissue cysts with fluorescently-tagged lectin (for immunofluorescence) [20]. DAPI counterstaining of nuclei visualized individual parasites in the tissue cysts, highlighting the hundreds of bradyzoites within each tissue cyst.

The presence of dopamine in tissue cyst-containing neural cells was confirmed by cytochemical staining and competition assays. Glyoxylic acid staining, a classic method for detection of dopamine-containing cells by chemical reaction of glyoxylic acid with dopamine to produce a fluorescent product [17], was applied. Interestingly, the tissue cyst infected cells fluoresced blue and white, with the entire cell body of the infected cell displaying fluorescent staining (Fig. 2A). Staining of the encysted parasites within cells and neural cell nuclei are black due to the presence of parasite and host nuclear chromatin. The lack of cytosolic staining in the immunofluorescent images with dopamine antibody are likely to be due to saturation of the image with the very intense cyst staining. The specificity of the dopamine antibody was confirmed by competition assays. Primary dopamine antibody staining of tissue sections was performed in the presence of exogenous dopamine followed by secondary staining with fluorescein labelled antibody. This eliminated staining as visualized by loss of fluorescence (Fig. 2B). In contrast, addition of exogenous serotonin (another catecholamine neurotransmitter) did not disrupt staining with the dopamine antibody. This verifies that the dopamine antibody is detecting dopamine. It remains possible that the dopamine antibody is also detecting the metabolic precursor to dopamine, L-DOPA, although manufacturer (Abcam) tests show a >400-fold higher affinity for dopamine compared to L-DOPA using conjugates to BSA. Competition assays exhibited some decrease in staining with exogenous L-DOPA although this was not quantifiable (data not shown).

### *T. gondii* infected cells release high amounts of dopamine

To assess whether the dopamine detected in *T. gondii* tissue cysts could affect neurotransmission, the effect of *T. gondii* infection on dopamine release from dopaminergic neural cells *in vitro* was determined. PC12 cells were utilized as this cell line is the most commonly used *in vitro* model of dopaminergic neurons. Dopaminergic PC12 cells were infected with *T. gondii* parasites incubated under conditions that induce differentiation, and dopamine content and release were monitored by HPLC-ED. Conditions were used (as described in the Materials and Methods) for stage conversion of tachyzoites (the rapidly dividing stage of *T. gondii*) into the tissue cyst stages (ie. bradyzoites) with alkaline pH and decreased CO_2_ content as described by others [4].

**Figure 3:**
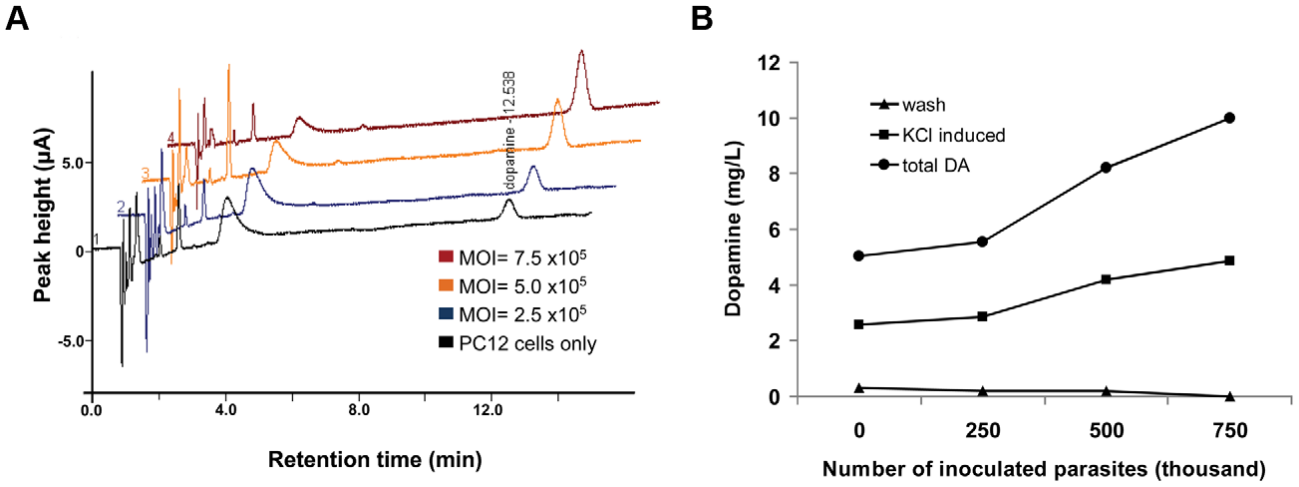
Elevated dopamine from *T. gondii* infected dopaminergic cells. (A) Overlay of HPLC-ED chromatograms derived from PC12 cells DA release assay, where cells were infected with increasing numbers of induced tachyzoites. PC12 cells are the classic dopaminergic neuron model since they contain all the machinery for dopamine synthesis, packaging and release. Equivalent numbers of cells were infected with *T. gondii* (brown, 7.5×10^5^; yellow, 5×10^5^; blue, 2.5×10^5^; and black, control) and incubated for 5 days followed by assaying DA release in high K+buffer. Increased dopamine was released from infected cultures. The amount of dopamine released is correlated with number of parasites in the culture. The experiment was repeated several times (n = 4) with a representative experiment shown. (B) Graph of dopamine released from the K+ induced cultures (squares) described in A. The total dopamine measured in each of the cultures is shown (circles). The dopamine measured in the low KCl wash buffer for each culture is also plotted (triangles).

The total dopamine in infected PC12 cultures was measured to determine whether infection increases the amount of dopamine synthesized in dopaminergic cells. Cultures were infected with different numbers of alkaline-induced *T. gondii* and total dopamine was quantitated by HPLC-ED following washes with low KCl buffer. We found that infected cultures accumulated significantly greater levels of dopamine and the increase correlated with infection rate (Fig. 3). Infection led to greater than three-fold increase in total dopamine content compared to mock-treated, uninfected cells.

Dopamine release assays were performed with cultures of *T. gondii*-infected PC12 cells to assess effects of infection on dopamine signalling. The cultures infected with different numbers of alkaline-induced *T. gondii* were induced to release dopamine with potassium as K+ causes release of dopamine in vesicles following methods in other studies [18]. As a result of infection, dopamine release increased in infected cultures in a dose-dependent manner with the number of parasites in the culture correlating with the amount of dopamine released (Fig. 3). Dopamine release in infected cells was up to 350% greater compared to dopamine release in uninfected cells. Dopamine release was specific for high KCl induction since wash buffer (Fig. 3B) and media alone (data not shown) did not induce the release of detectable amounts of dopamine in infected or uninfected cultures. The low KCl wash ensures that the dopamine released is induced by potassium and not due to dopamine released by cell lysis of infected cells. The dopamine release reported here is the minimum amount increased by *T. gondii* infection as less than or equal to half of the cells in the cultures were infected. Normalizing for the infection rate results in a seven-fold increase in dopamine release in infected cells relative to uninfected PC12 cells.

Taken together, an increase in dopamine content and an increase in dopamine release were observed in neural cells as a direct response to *T. gondii* infection. The enhanced dopamine release observed in infected cells in this study is likely to be an underestimate of the effect on dopamine release *in vivo*, cultured parasites contain few bradyzoites per vacuole compared to brain tissue cysts that contain hundreds of bradyzoites.

### Tyrosine hydroxylase is expressed in bradyzoites

**Figure 4:**
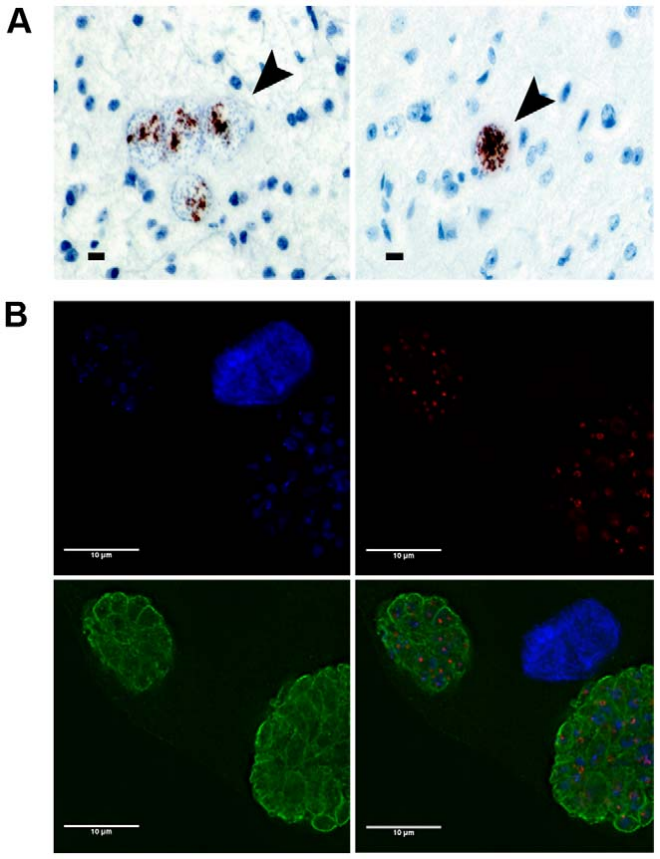
Dopamine enzyme tyrosine hydroxylase in intracellular *T. gondii*. (A) Immunohistochemical localization of tyrosine hydroxylase (TH) in brain sections of chronically-infected mice with commercial antibody and horseradish peroxidase labelling. Tissue cysts are visible as brown circular structures (left, four cysts, and right, single cyst, highlighted with arrowheads). (B) TH in intracellular parasites *in vitro*. Alkaline-induce parasite cultures were probed with anti-tyrosine hydroxylase antibody (green), RFP-GRASP (red), and DAPI (blue) shown separately and as a composite image. Scale bars on all images are 10 µM.

Tyrosine hydroxylase is the rate-limiting enzyme in dopamine biosynthesis. Tyrosine hydroxylase localization in the brain sections of mice chronically infected with *T. gondii* was determined to examine the expression of this crucial enzyme in infected neural cells. Significant levels of tyrosine hydroxylase were localized *within T. gondii* tissue cysts in the brain sections of infected mice (Fig. 4). As expected, tyrosine hydroxylase was also found in the cytosol of neurons in the expected areas of the brain in both infected and uninfected mice (data not shown). Staining was not apparent in control sections that were treated with only secondary antibody. It is intriguing that both tyrosine hydroxylase and dopamine staining were localized in the tissue cysts of infected mouse brains, displaying similar staining patterns (Figs. 1, 4). Thus, the rate limiting enzyme for dopamine synthesis and the product itself were both found *in T. gondii* tissue cysts in the brain.

It is possible that the tyrosine hydroxylase expression observed within the tissue cyst could be either the *T. gondii*-encoded tyrosine hydroxylase or neuronal tyrosine hydroxylase that has been imported from the host. *T. gondii* has complex interactions with its host cell and co-ops several host proteins (e.g. calpains), and hence could potentially import neuronal tyrosine hydroxylase into the tissue cyst [21].

Alternatively, *T. gondii* could provide an enzyme with tyrosine hydroxylase activity. We previously described a *T. gondii* encoded tyrosine hydroxylase that could be expressed in the brain tissue cysts [9]. *T. gondii* has two copies of the tyrosine hydroxylase gene encoding nearly identical proteins (97.5%) with one gene induced in bradyzoite-stage parasites. The parasite tyrosine hydroxylase has a high degree of homology (53% identity) with mammalian tyrosine hydroxylases. Unique for tyrosine hydroxylases, the parasite orthologue enzyme contains a putative signal sequence that could permit the enzyme to be trafficked to an organelle or secreted by *T. gondii*. Additionally, it was observed that the commercial tyrosine hydroxylase antibody used in these studies recognizes the *T. gondii* encoded orthologue, as well as the mammalian tyrosine hydroxylases (unpublished observations). Indeed, *in vitro* cultivated parasites under alkaline conditions that induce formation of bradyzoites bind commercial tyrosine hydroxylase antibody (Fig. 4B). The tyrosine hydroxylase antibody stains the parasitophorous vacuole and also stains the periphery of parasites.

**Figure 5:**
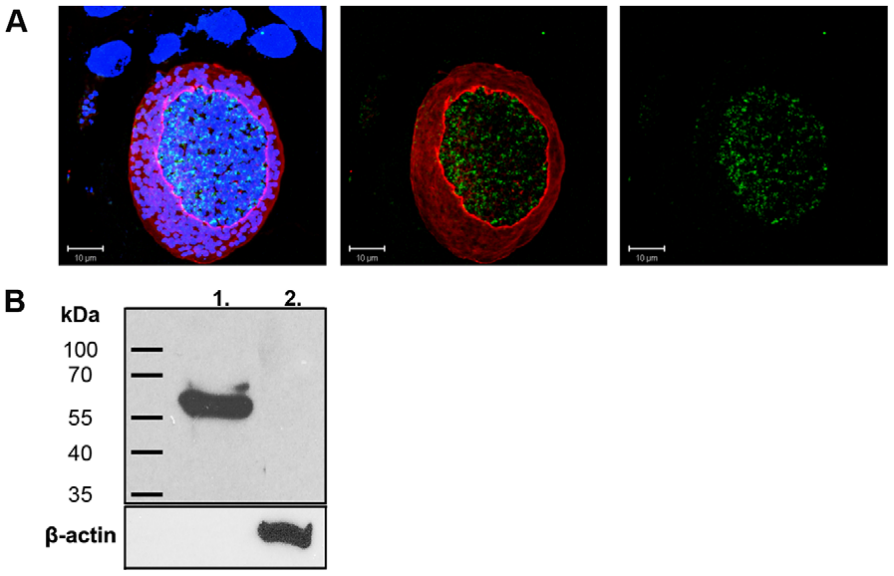
Expression of a parasite-encoded tyrosine hydroxylase in brain tissue cysts. (A) 3D projections of serial images of *T. gondii* tissue cysts within brain sections were triple stained with *T. gondii* encoded tyrosine hydroxylase (TgTH) antibody (green), DAPI (blue), and lectin (red). The panels (from left to right) show all three channels, the lectin and antibody, and TgTH antibody alone (green). Staining was not apparent in control sections that received only secondary antibody (data not shown). DAPI identified neuronal cells and the individual bradyzoites within the tissue cyst and lectin stained the surface of the cyst. (B) Western analysis for specificity of the custom antibody for TgTH. Recombinant protein from Δ29TgAaaH2 [9] and mouse brain were probed with TgTH antibody. No bands were detected in uninfected mouse brain. β-actin was used as a loading control.

To specifically identify parasite-encoded tyrosine hydroxylase within brain tissue, a custom antibody for *T. gondii* tyrosine hydroxylase (TgTH) was developed. The target sequence of this custom antibody is located in the amino-terminal domain of TgTH, which is unique and divergent from mammalian tyrosine hydroxylases. *T. gondii* tyrosine hydroxylase was localized within tissue cysts in neural cells in chronically-infected mouse brains (Fig. 5A). Staining was only detectable in tissue cysts in infected neurons. Western analysis was performed to validate the specificity of the TgTH antibody. The antibody recognized recombinant TgTH but did not bind mouse brain proteins, confirming the specificity of this antibody for *T. gondii* tyrosine hydroxylases (Fig. 5B). Hence the intense dopamine antibody staining and TgTH are both found in *T. gondii* brain tissue cysts.

## Discussion

Changes in behavior of the intermediate host that could lead to increased transmission of a parasite to its definitive host are likely to be positively selected as these changes would provide a significant benefit in completion of the parasite's life cycle. *T. gondii* induces behavioral alterations in infected rodents that would facilitate the transmission of the parasite to its definitive feline host, however, the mechanism responsible for these changes remains unclear. Our study provides a mechanism for these changes. Previous studies showing that anti-dopaminergic drugs can prevent the development of the behavior changes in rodents suggest that dopamine regulation altered by *T. gondii* infection of mammals [7].

The altered behavior may be a direct effect or an indirect effect of *T. gondii* infection. In this study, significant levels of dopamine was detected by immunohistochemistry in *T. gondii* tissue cysts in the brain (Fig. 1), as well as, increased dopamine release from dopaminergic cells infected with *T. gondii* (Fig. 3). Based on these novel findings, this is the first study to suggest that a parasite can directly alter dopamine signalling to mediate host behavior changes. These results provide a potential mechanism for *T. gondii*-induced host behavioural changes.

In our study, localizing the changes in dopamine metabolism during infection was crucial, as the location of dopamine metabolic changes in the brain is likely to be a critical factor for its effect on host behavior. Encysted *T. gondii* have been observed in functional neurons with intact synapses [22], [23]. Tissue cysts have been detected throughout the brain, although higher percentages of cysts were reported in the amygdala and nucleus accumbens [5], [24]. These limbic brain regions are well known to contain dopamine that plays important functions in the control of movements (basal ganglia), reward to stimuli, pleasure, dependency (nucleus accumbens and hippocampus), motivation and cognition, and species and stimuli specific fear (amygdala). Altered dopamine levels induced by *T. gondii* in tissue cysts in these regions of the brain could have significant harmful consequences on a variety of brain functions, possibly leading to an array of behavioral changes and possible neurological malfunctions.

The observed intense dopamine staining within the *T. gondii* tissue cysts in brains was unexpected. Dopamine in neurons is synthesized in the cytosol, packaged into vesicles, and transported along axons [25]. Thus, dopamine staining in neurons is primarily detected within vesicles. Indeed, cytosolic dopamine can induce cell apoptosis if it is not properly packaged into vesicles [26]. Packaged dopamine in neurons is rapidly transported away from the cell body to the axon terminal. In our brain sections, any dopamine released from the cyst into the cell body of the neuron would be packaged and transported by the efficient dopaminergic vesicle transport along axons. This may explain the apparent lower level of dopamine in the host cell body compared to the tissue cyst (Figs. 1, 2). Alternatively, the observed staining by the dopamine antibody could be due to detection of L-DOPA within the *T. gondii* tissue cyst that escapes the cyst and is metabolised into dopamine in the host cytosol. This interpretation is coherent with the observed cytosolic staining of infected neurons using glycoxylic acid that yields a specific product with dopamine (Fig. 2). The parasite provides tyrosine hydroxylase, the rate-limiting enzyme in dopamine synthesis but the source of DOPA decarboxylase required for conversion of L-DOPA to dopamine needs further investigation. DOPA decarboxylase present in the cytosol of dopaminergic neurons could provide this enzyme. A hypothetical nutrient pore expressed in the parasitophorous vacuole membrane of *T. gondii* tachyzoites that permits the passage of metabolites (<1300 Da) from the host cell cytosol into the parasitophorous vacuole could allow passage of small compounds from the vacuole into the host cytosol [27]. If the pore is expressed in bradyzoites then it could provide a means for dopaminergic metabolites (L-DOPA, dopamine) to exit the vacuole and enter the host cytosol where L-DOPA would be converted to dopamine by cytosolic DOPA decarboxylase and dopamine would be packaged into secretory vesicles. The generation of *T. gondii* mutants that will provide a conclusive dissection of the role of the parasite's tyrosine hydroxylase in the dopamine synthesis and release are in progress, but with either site of dopa decarboxylase action, the increased dopamine metabolism has important implications on the host neurochemistry.

In addition to dopamine, neurotransmitters such as serotonin and glutamate need to be considered in *T. gondii*-induced behavioral changes. Prior studies have proposed that the host immune response to *T. gondii* infection may lead to altered neurotransmitter levels [28]. Immunocompetent hosts control chronic *T. gondii* infection with a T-lymphocyte–driven defense [29]. Infection of mice with *T. gondii* elicits a dominant Th1 response involving interferon-gamma (IFN-γ), interleukin-12 (IL-12), IL-18, and tumor necrosis factor alpha (TNF-α). TNF-α induction has a serious impact on *T. gondii* induced pathology at early stages of infection. Th2-associated cytokines, such as IL-4 and IL-10, appear relatively late after infection and may limit immune pathology. To resolve acute infection, IFN-γ induces indoleamine 2,3-dioxygenase (IDO) release, resulting in tryptophan degradation and kynurenic acid accumulation [30]. Tryptophan depletion is thought to be responsible for suppression of the growth of the acute stage tachyzoites. Changes in serotonin levels were not observed in mice with *T. gondii* chronic infections although there may be localized undetected changes [19]. Kynurenic acid accumulation in the CNS could potentially alter dopamine metabolism due to its NMDA antagonistic properties [12]. Thus, the host immune response to *T. gondii* infection could contribute to alterations in neurotransmitter levels that could affect behaviour in conjunction with the increased dopamine mediated by the parasite. Further studies are essential to investigate these possibilities.

Behavioral changes associated with *T. gondii* infection may contribute to serious neurological disorders in humans. Several studies have observed an association between *T. gondii* seroprevalence with schizophrenia [10], [13]. Since *T. gondii* infection has been found to last throughout the lifetime of the host, seroprevalence is likely to reflect chronic infection [4]. Dopamine dysregulation is proposed to play a central role in schizophrenia, potentially in combination with glutamate metabolism. How dopamine dysregulation plays a role in schizophrenia, however, is still unknown. The principal antipsychotic drug that has been used to treat schizophrenia, dopamine antagonist haloperidol, can also block the development of behavior changes in *T. gondii* infected rodents. It is possible that the increased dopamine accumulation and release observed during *T. gondii* infection may contribute to *T. gondii* associated schizophrenia. Dopamine metabolite concentrations have been inversely correlated with gray matter volume in schizophrenia patients, and recent MRI evidence found that the majority of volume reduction is in those patients seropositive for *T. gondii*, suggesting that *T. gondii* infection leads to an increase in dopamine metabolite concentrations [31], [32]. It would be of interest to analyze the ability of other pathogens associated with schizophrenia, and other neurological disorders, to directly alter dopamine metabolism to see if other pathogens have this ability or if this phenomena is unique to *T. gondii*.

Malfunctions of dopamine metabolism have a serious impact on human behavior. Dopamine dysfunction has been associated with a variety of neurological disorders including schizophrenia, attention deficit hyperactivity disorder, tic disorders, Tourette's syndrome, and dyskinesias. The novel findings of this study, that demonstrate *T. gondii's* ability to directly alter dopamine levels will not only help to better understand the relationship between schizophrenia and *T. gondii* seroprevalence, but these findings may be critical for understanding the mechanism(s) involved in a variety of pathogen-associated neurological disorders [10], [13]. Thus, it is crucial to determine if other pathogens associated with neurological disorders also have the ability to directly alter dopamine levels. It is also critical to determine the possible contributions of *T. gondii* infection to other dopamine-related diseases [33], [34].
